# Non-Starch Polysaccharides in Durum Wheat: A Review

**DOI:** 10.3390/ijms21082933

**Published:** 2020-04-22

**Authors:** Ilaria Marcotuli, Pasqualina Colasuonno, Yves S. Y. Hsieh, Geoffrey B. Fincher, Agata Gadaleta

**Affiliations:** 1 Department of Agricultural and Environmental Science, University of Bari ‘Aldo Moro’, Via G. Amendola 165/A, 70126 Bari, Italy; pattybiotec@yahoo.it; 2 Division of Glycoscience, Department of Chemistry, School of Engineering Sciences in Chemistry, Biotechnology and Health, Royal Institute of Technology (KTH), SE106 91 Stockholm, Sweden; yvhsieh@kth.se; 3 ARC Centre of Excellence in Plant Cell Walls, School of Agriculture, Food and Wine, University of Adelaide, Waite Campus, Glen Osmond, SA 5064, Australia; geoffrey.fincher@adelaide.edu.au

**Keywords:** durum wheat, fibre content, arabinoxylan, (1,3, 1,4)-β-glucans

## Abstract

Durum wheat is one of most important cereal crops that serves as a staple dietary component for humans and domestic animals. It provides antioxidants, proteins, minerals and dietary fibre, which have beneficial properties for humans, especially as related to the health of gut microbiota. Dietary fibre is defined as carbohydrate polymers that are non-digestible in the small intestine. However, this dietary component can be digested by microorganisms in the large intestine and imparts physiological benefits at daily intake levels of 30–35 g. Dietary fibre in cereal grains largely comprises cell wall polymers and includes insoluble (cellulose, part of the hemicellulose component and lignin) and soluble (arabinoxylans and (1,3;1,4)-β-glucans) fibre. More specifically, certain components provide immunomodulatory and cholesterol lowering activity, faecal bulking effects, enhanced absorption of certain minerals, prebiotic effects and, through these effects, reduce the risk of type II diabetes, cardiovascular disease and colorectal cancer. Thus, dietary fibre is attracting increasing interest from cereal processors, producers and consumers. Compared with other components of the durum wheat grain, fibre components have not been studied extensively. Here, we have summarised the current status of knowledge on the genetic control of arabinoxylan and (1,3;1,4)-β-glucan synthesis and accumulation in durum wheat grain. Indeed, the recent results obtained in durum wheat open the way for the improvement of these important cereal quality parameters.

## 1. Introduction

Cultivated wheats (*Triticum spp.*), which include several species of the family Poaceae, originated in the Levant region of the Near East [1] and are now cultivated worldwide.

Cultivated wheats are divided in three main groups based on their genome composition, which can be diploid, tetraploid or hexaploid. About 95% of the wheat grown worldwide is hexaploid (AABBDD genomes) bread wheat (*Triticum aestivum*). The remaining 5% is the tetraploid (AABB genomes) durum wheat (*Triticum turgidum*). The latter is often called pasta wheat to reflect its major end-use (pasta, bulgar, couscous and some bread flours) [2] and is typically grown in the Mediterranean area. Here we will refer to it simply as durum.

In the Gran Maghreb and East Africa durum grain is a staple human food in rural areas and its straw is a critical component of domestic animal diets. In Europe, but also in most other continents, durum products like pasta have been associated with modernising of societies and increased female employment, because durum products are perceived as “healthy foods” that can be rapidly prepared. In rural communities and in poorer regions of cities, durum products are mostly consumed alone or with minor additions of simple foods, and durum grains therefore represent a key intake of nutrients in the diets of smallholder farmers and undernourished people [3].

Durum grains contain a storehouse of nutrients essential for the development of the young seedling following grain germination, but these nutrients are also beneficial in the human diet. The main components of durum grains include starch (70.2%), proteins (12.2%), lipids (1.9%), fibre (1.6%) and minerals (1.6%), with varying water content. Durum kernels have a high carbohydrate content and high antioxidant levels (such as carotenoid pigments), together with higher vitamin, sodium, potassium, calcium and magnesium contents, compared with other cereals (Table 1).

Thanks to the nutritional properties of cereal grains and due to the modern revolution in the perception of food, durum has gained popularity within health and wellness circles as sources of nutrients and energy that are associated with a number of powerful health benefits, as mentioned above.

## 2. Dietary Fibre and Nutritional Aspects

Exploitation of natural variation in bioactive components in durum grain, such as levels of minerals, resistant starch, antioxidant compounds like carotenoids [4], grain protein and dietary fibre [5], has been a key target for genetic improvement and for the development of desired cultivars with better nutritional value. Durum is one of the principal sources of fibre for human and livestock nutrition, in particular with respect to arabinoxylan and, to a lesser extent, in (1,3;1,4)-β-glucan. Over a decade of intensive research, the role of dietary fibre in lowering the risk of diet-related chronic diseases has been established [6,7].

Dietary fibre is defined as the edible part from plants which are resistant to enzymatic digestion in the small intestineand include cellulose, non-cellulosic polysaccharides (hemicellulose, pectic substances, gums, mucilages) and non-carbohydrate component. The species with higher fibre content are cereals, which contribute to up to 50% of the human fibre intake, followed by vegetables, fruit and nuts, with 30–40%, 16% and 3%, respectively, of the fibre contribution [8,9].

A large clinical study by the European Prospective Investigation into Cancer and Nutrition (EPIC) showed that dietary fibre consumption reduces the risk of colon cancer and diverticular diseases [10,11,12]. In addition, it has been demonstrated that the structural properties of non-starch polysaccharides are strongly linked to food digestibility, bulking and fermentability [13,14,15]. The key non-starch polysaccharides of cereal grains are heteroxylans (predominantly arabinoxylans) and (1,3;1,4)-β-glucans, both of which are long, highly asymmetrical molecules and therefore form solutions of high viscosity. The solubility of these polysaccharides is influenced by the degree of arabinosyl substitution of the xylan backbone in the case of the arabinoxylans [15], and by the distribution of (1,3)- and (1,4)-linkages in the case of the (1,3;1,4)-β-glucans [14].

Thus, the presence of dietary fibre in the small intestine increases the viscosity of the bulk and therefore slows down enzyme-mediated starch breakdown, both through slower diffusion of α-amylases and other starch degrading enzymes in the contents of the small intestine and through slower diffusion of degradation products back to the intestinal wall. This, in turn, prolongs the food absorption process and slows the rate of glucose release into the bloodstream after meals. The attendant reduction in glycaemic index provides benefits to people with type II diabetes [16].

In the large intestine, dietary fibres are fermented to various intermediary metabolites, including the short-chain fatty acids acetate, propionate and butyrate, which are likely candidates to reduce the risk of colorectal cancer [17]. Arabinoxylan and (1,3;1,4)-β-glucan exert their beneficial impact on human health not only by increasing the viscosity of digesta, but also by altering of composition of microbial flora in the human gut and hence in the relative abundance of specific short chain fatty acids and other metabolites. Thus, these polysaccharides have been recognised as potential pharmaceutical preventative agents of diet-related chronic diseases, when taken at appropriate doses.

In addition to carbohydrate components, the arabinoxylans possess phenolic moieties, such as ferulic acid and *p*-coumaric acid, both of which are ester-linked to the 5(C) atom of arabinofuranosyl residues of the arabinoxylan chain. The presence of these substituents may lead to covalent cross-linking of arabinoxylan chains [18] and can therefore influence the solubility of arabinoxylan. Some reports suggested that ferulic acid released by esterase activity in the digestive system can act as an antioxidant that lowers the risk of colorectal cancer [17,19]. Therefore, intensive research activities have been invested in selecting cereal traits that have high levels of phenolic components [20].

In the last few years, thanks to the growing interest in renewable materials as an alternative to fossil resources for the production of commodity chemicals and energy, plant residues have been recognised as an extraordinarily large source of fermentable sugars, which can be used for biofuel production [21,22,23], and residues from cereal crops and from downstream processing of cereals contain large amounts of heteroxylans.

## 3. (1,3;1,4)-β-Glucan in Durum Wheat: Structure and Biosynthesis

**Figure 1:**
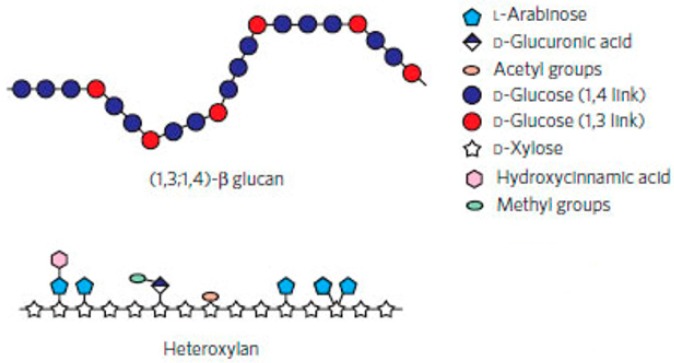
Structures of non-starch polysaccharides in plants. The backbone of (1,3;1,4)-β-glucan is based on (1,4)-β-linked monosaccharides, whereas the backbone of arabinoxylan shows L-arabinose units attached to O-2 or O-3 of D-xylose residues. Some arabinose residues are esterified at O-5 by a hydroxycinnamic acid. D-Glucuronic residues were reported as substituents at O-2 of the xylose residues such as acetyl groups. Figure redrawn from Burton et al., 2010 [22].

The (1,3;1,4)-β-glucans are abundant in cell walls of the Poaceae and represent one example in which heterogeneity in fine structure is essential for function in the plant cell wall [24]. The (1,3;1,4)-β-glucans consist of unsubstituted β-glucopyranosyl units joined by varying proportions of (1,3)- and (1,4)-linkages (Figure 1). Within the grasses, barley (*Hordeum vulgare*), oat (*Avena sativa*) and rye (*Secale cereale*) grain are rich sources of (1,3;1,4)-β-glucans, while wheat (*Triticum aestivum*), rice (*Oriza sativa*) and maize (*Zea mais*) have lower (1,3;1,4)-β-glucan contents [25,26].

The ratio of the (1,4)- to (1,3)- linkages in grain varies between species, from 2.5:1 to 3.4:1 in durum [27], from 2.9:1 to 3.4:1 in barley, from 1.8:1 to 2.3:1 in oats and 2.7:1 in rye [28]. As adjacent (1,3)-linkages are rare or absent, these single (1,3)-linkages are separated by mostly two or three (1,4)-linkages, but also by longer regions of up to 10 (1,4)-linkages [29]. The common groups of (a) one (1,3)-linkage and two (1,4)-linkages (degree of polymerisation of three (DP3)) and (b) one (1,3)-linkage and three (1,4)-linkages (DP4) can be used to predict the solubility of the polysaccharide [30]. As the groups of two or three (1,4)-linkages are arranged at random along the backbone of the polysaccharide [31], the single (1,3)-linkages, which cause a kink in the symmetry of the molecule, are also arranged at irregular intervals. For this reason, the molecules will not align over extended regions and can remain in solution when their degree of polymerisation (DP) exceeds 1000 [32]. It should be noted however that the ratio of (1,4)- to (1,3)-linkages varies both within a species and between different species and this accounts for the varying solubilities of this polysaccharide. The asymmetrical conformation is crucially involved in function of the gel-like matrix phase of the cell wall, which allows the wall to be supported but at the same time it remains flexible, pliable and porous to permit transfer of water, nutrients and other small molecules [25]. As mentioned above, the same asymmetry is responsible for the high viscosity of (1,3;1,4)-β-glucan when it is extracted from cereal grains in the alimentary tract of monogastric animals and hence for the beneficial effects on human health and nutrition [33].

The molecular mechanism of (1,3;1,4)-β-glucan synthesis has not yet been defined in unequivocal terms. There are several possibilities. Firstly, there is some evidence that the CslF and CslH (1,3;1,4)-β-glucan synthase enzymes can insert both types of linkages during chain elongation [26]. Secondly, it has been suggested that a pair of enzymes is required, one of which inserts (1,3)-linkages and the other (1,4)-linkages. In one case this could involve an enzyme complex of, for example, a CslF enzyme that inserts single or multiple (1,4)-linkages and a glucan-synthase-like (GSL) enzyme that inserts single (1,3)-linkages. Alternatively, (1,4)-β-oligoglucosides (cellodextrins) could be synthesised by the CslF enzyme and these oligoglucosides could subsequently be linked by a separate enzyme via a (1,3)-linkage [34]. Thirdly, it is possible that the CslF enzyme produces only (1,4)-β-glucan chains and that the (1,3)-linkages are introduced by transglycosylation of that (1,4)-β-glucan chain; a candidate enzyme for catalysing such a transglycosylation reaction is the xyloglucan endotransglycosylase (XET) [24]. However, a precise definition of the detailed mechanism of (1,3;1,4)-β-glucan chain assembly awaits a detailed three-dimensional structure of one of the (1,3;1,4)-β-glucan synthase enzymes.

## 4. Physicochemical and Functional Properties of (1,3;1,4)-β-Glucans

The (1,3;1,4)-β-glucan structure discussed above influences overall molecular conformation and biological function. At very low or very high DP3:DP4 ratios the molecular kinks imposed by the (1,3)-β-glucosyl residues will become more regularly spaced in the polysaccharide, which, in turn, will become less soluble and less suitable for gel formation in the matrix phase of the wall. The conformational irregularity of (1,3;1,4)-β-glucans in cell walls therefore appears to be a feature confined to the polysaccharides of Poaceae. Even within the Poaceae family, there are significant differences in the fine structures of the (1,3;1,4)-β-glucans. The conformational regularity or irregularity of (1,3;1,4)-β-glucans define its properties and physicochemical behaviour in the cell wall matrix. As noted above, the irregularly spaced molecular kinks produce an asymmetrical shape that cannot easily align or aggregate into fibrils, but rather is soluble and, through limited intermolecular junction zone formation, is capable of producing a gel-like material offering some structural support for the wall, combined with flexibility and porosity [25]. Such interactions in the context of the cell wall can be influenced by other factors, including associations with other polysaccharides or proteins. Recent observations underline that different isoforms of a single enzyme family can direct the synthesis of wall polysaccharides with different structures and thus different physicochemical properties [35].

In the Poaceae, (1,3;1,4)-β-glucans are often associated with growing cells [36], are essentially absent in mature vegetative tissues [37,38,39], and are generally found in expanding cells of organs such as the coleoptile [39,40] and some of the vascular and fibre cells of the leaf, suggesting a structural role in secondary cell walls [41]. They may or may not be present in the starchy endosperm of grain. Thus, (1,3;1,4)-β-glucans are not found in all cell walls of all tissues in the Poaceae and hence are not essential for wall structure or function [42].

In the walls of the starchy endosperm, which do not exhibit secondary thickening, the (1,3;1,4)-β-glucans can be present at high concentrations and can contribute up to 18% of the total glucose stored in the grain [43]. Thus, they clearly participate as storage polysaccharides in the grain and are mobilised when the grain germinates. This is clearly the case in the starchy endosperm of *Brachypodium distachyon*, in which cell walls contain up to 45% (1,3;1,4)-β-glucans [36,44] and where this polysaccharide replaces starch as the major storage polysaccharide in the grain.

The (1,3;1,4)-β-glucans, thanks to their solubility and viscosity in aqueous media and to their ability to alter the composition of large intestine microbiota, improve the health and efficiency of the human digestive system. In addition, there is a great potential end-use of (1,3;1,4)-β-glucans as functional food ingredients to improve mouthfeel of low-fat dairy products [33].

## 5. (1,3;1,4)-β-Glucan Content and QTL Regions in Durum Wheat

The (1,3;1,4)-β-glucan content in durum whole grain is measured by the Mixed-Linkage (1,3;1,4)-β-glucan Assay Kit (Megazyme International Ireland Ltd., Wicklow, Ireland) based on the accepted method of McCleary and Codd [45]. The kit includes an industrial standard for barley (4.1% (1,3;1,4)-β-glucan). As mentioned before, (1,3;1,4)-β-glucans are a relatively minor component of durum wheat grain, so investigations of durum genotypes with higher amounts [46,47,48] or wheat-related species such as *Aegilops* ssp. have been carried out [46,49,50,51,52,53,54,55] for further genetic-molecular analysis and for the analysis of possible interspecific gene transfer.

**Figure 2:**
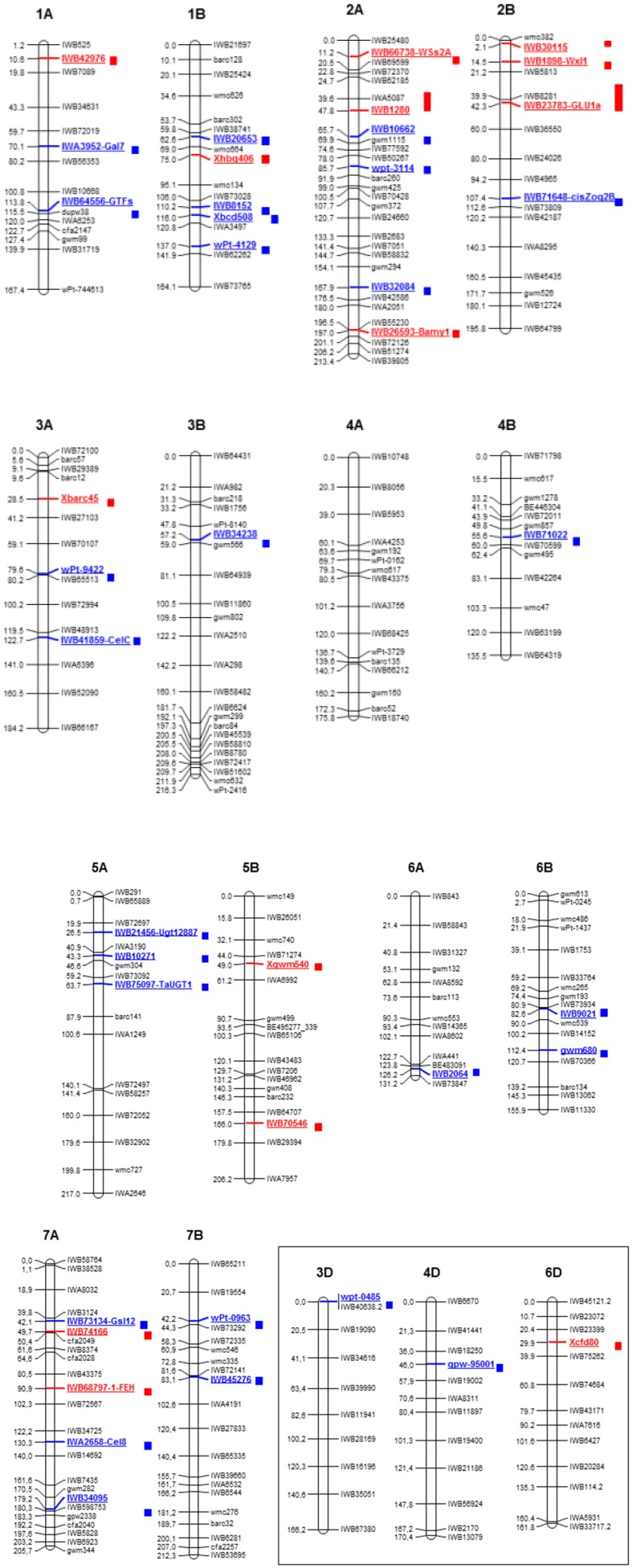
Schematic representation of durum wheat chromosomes (A and B genomes) from the consensus map [57] and chromosomes 3D, 4D and 6D from bread wheat with quantitative trait loci (QTL) summary for (1,3;1,4)-β-glucan and arabinoxylan trait detected in references from Table 2 and Table 3. Markers, on the right chromosome side, are reported every 20 cM approximately. cM distances are indicated on the left side of the bar. Red solid bars indicate the QTL confidence interval regions for (1,3;1,4)-β-glucan, while blue bars designated the arabinoxylan QTL.

Here we report data from marker trait association for the identification of QTL associate to (1,3;1,4)-β-glucan content in durum kernel and controlling the trait in recombinant inbred lines of a durum population through a classical association mapping approach [48,56] and genome-wide association studies (GWAS) carried out on a collection of tetraploid wheats [47]. A total of 14 QTL have been identified and are distributed across most durum chromosomes (Figure 2, Table 2), with the highest number on chromosome group 2 [47,48]. Some of the markers associated with the trait are located in the QTL regions containing genes encoding for glycosyl transferase (GT) and glycosyl hydrolase (GH) enzymes. For example, QTL on chromosome 2A were associated with alleles of genes encoding starch synthase II (*WSs2A*) and β-amylase, while QTL on 2B were associated to the β-glucosidase (*GLU1a*) and the (1,3;1,4)-β-glucanase isoenzyme II (*Glb2*). Even though QTL were reported on durum chromosome group 2, no associations were detected with the *CslH* gene on this chromosome. This could be due to the lack of polymorphic SNP markers in the gene region and/or in the position of the QTL and the gene. In the Triticum collection used for GWAS we found two associations on chromosome 2A (at 11.2 and 197 cM, respectively) and one on chromosome 2B (at 14.5 cM), while in a RILs population we reported one QTL for (1,3;1,4)-β-glucan on chromosome 2A (35.8–48.0 cM) and two QTL on chromosome 2B (0.1–3.9 and 29.9–47.9 cM, respectively). Using these data, we tried to map the *CslH* gene on the consensus map of Maccaferri et al. [57]. We were able to locate the gene only on chromosome 2B at 100.5 cM, thanks to a co-migrant marker detected in the same contig of the gene, not close to the QTL reported above.

Similarly, none of the previously identified QTL have significant associations on chromosomes 7A and 7B with the *CslF6* gene that encodes a (1,3;1,4)-β-glucan synthase. In this case we failed to map the position of the gene on chromosome group 7 in durum. It is noteworthy that associations with isoamylase and (1,4)-β-xylan endohydrolase were reported on chromosome 7A [27].

Following a recent report that identified *CslJ* as a gene capable of directing (1,3;1,4)-β-glucan biosynthesis [58], we report for the first time in the current review the location of this gene in the durum material. Using the annotated sequence of cv. Svevo and the consensus map [57], we located *CslJ* on the chromosome 3 group and were able to map it on the B genome at 33.0–40.0 cM, far from the single QTL (at 97.2 cM) reported by Marcotuli et al. [47].

## 6. Candidate Genes for (1,3;1,4)-β-Glucan in Durum

The main gene families that control (1,3;1,4)-β-glucan synthases are members of the large family of *cellulose synthase* (*CesA*) [59,60,61,62,63] and *cellulose-synthase-like* (*Csl*) genes [58,64,65]. The *Csl* gene sub-families (designated as sub-families A to M) [58,66] consist of multiple genes, some of which are specific for eudicots (B, G, E and M subfamilies) while others are specific for monocotyledons (F, H and J subgroups) [58,65,67,68].

**Figure 3:**
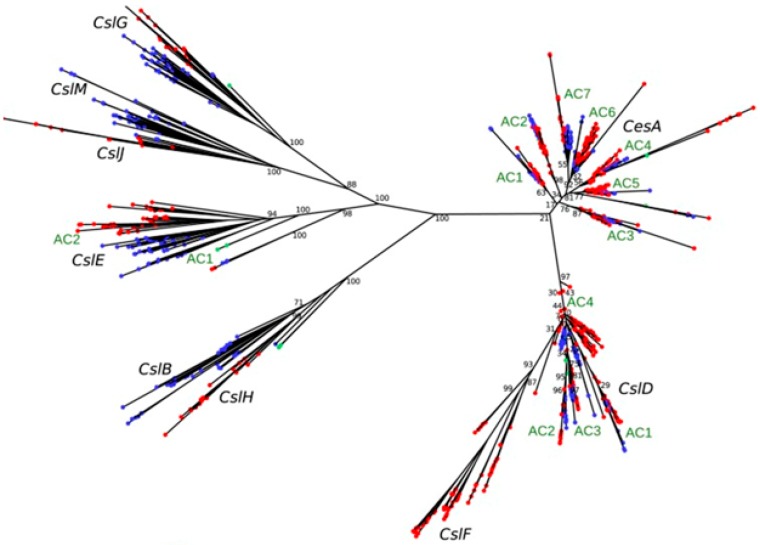
Cellulose synthase superfamily gene trees constructed using RAxML. Red external nodes indicate monocots, blue nodes indicate eudicots, and green nodes indicates the basal angiosperm *Amborella trichopoda*. Figure from Little et al., 2018 [54].

Recently, the phylogeny of the *Csl* genes was revised by Little et al. [58] (Figure 3). The data reported highlight that the *CslF* monocot subfamily is a sister group of the eudicot *CslD* sub-family [58,65], that the monocot *CslJ* genes are sisters to the *CslM* sub-family, and that the monocot *CslH* clade is sister to the *CslB* eudicot-specific sub-family.

Many studies reported the effect of the *CslF* gene in regulation of (1,3;1,4)-β-glucan accumulation in different tissues types [69,70]. Initial proof-of-function was obtained by insertion of the *OsCslF6* gene from rice *Arabidopsis*, which has no *CslF* genes and no (1,3;1,4)-β-glucan in its cell wall. It was demonstrated that (1,3;1,4)-β-glucan was deposited in the cell walls of the transgenic *Arabidopsis* plants, indicating that the inserted gene was capable of synthesising this polysaccharide [64]. Partial silencing the *CslF6* gene by dsRNAi resulted in barley grains that contained greatly reduced levels of (1,3;1,4)-β-glucan [71]. Furthermore, overexpression of *HvCslF6* in barley resulted in an increase of (1,3;1,4)-β-glucan content, accompanied by a dramatic decrease of starch [68].

Different genes of the *CslF* sub-family were identified in barley and mapped on different chromosomes: *HvCslF3*, *HvCslF4*, *HvCslF8*, *HvCslF10* are on chromosome 2H; *HvCslF9* is on chromosome 1H; *HvCslF6* is on chromosome 7H [72]. The effects of each barley chromosome on the (1,3;1,4)-β-glucan content were analysed in a complete series of wheat-barley addition lines, and although a slight variation in the trait was attributable to the presence of chromosomes 1H, 2H, 6H and 7H, levels of the polysaccharide in the addition lines were much lower than in barley [50].

**Figure 4:**
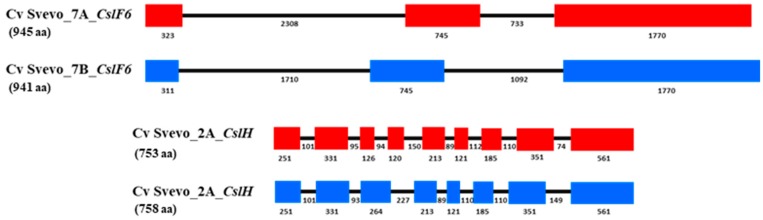
Schematic representation of *CslF6* and *CslH* gene structures in durum wheat (A and B genomes). Intron and exon sizes are shown as well as the whole gene (in brackets the total length). Figure redrawn from Marcotuli et al., 2018 [69].

Few studies have been carried out on *CslF6* in durum. In our recent study on the isolation and characterisation of the *CslF6* gene from durum cv. Svevo [73] (Figure 4, we showed that *CslF6* transcript abundance in developing durum grain peaked at 21 dap, with significant and positive correlation between (1,3;1,4)-β-glucan content and *CslF6* gene expression at 21 dap and 40 dap [73].

Members of the *CslH* gene family have also been identified as candidates for (1,3;1,4)-β-glucan synthesis through transformation of *Arabidopsis* with the *HvCslH* gene from barley [34]. In durum we found that the *CslH* gene was expressed mainly in durum endosperm at different developmental stages, specifically at 28 days after pollination (DAP) [73]. Different data were obtained in barley grain, where *HvCslH* transcript level showed a low amount through endosperm developmental stages with a peak of expression at 4 DAP [34].

Similarly, members of the *CslJ* clade are able to mediate the synthesis of (1,3;1,4)-β-glucans as demonstrated by Dimotroff et al. [74] through the expression of the gene in *Nicotiana benthamiana*. At this stage, the role of this gene in (1,3;1,4)-β-glucan synthesis in durum has not been examined.

## 7. Arabinoxylan in Durum Kernels: Structure and Biosynthesis

**Figure 5:**
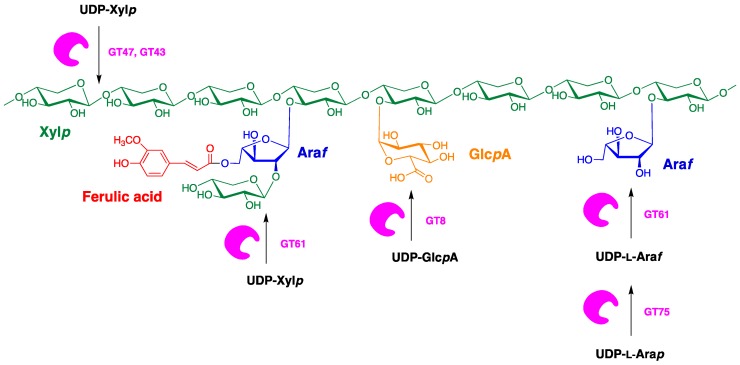
Predicted roles of glycosyltransferase (GT) gene families in arabinoxylan biosynthesis.

Arabinoxylans of cereal grains consist of a backbone of β-d-xylopyranosyl units (Xyl*p*), some of which are substituted with single α-l-arabinofuranosyl (Ara*f*) at O-3 and/or O-2. Thus, the Xyl*p* residues can be monosubstituted or disubstituted with Ara*f* residues (Figure 5). In wheat and rye arabinoxylans, only small amounts of O-2 substitution occur [75]. The molecular structure of the xylan backbone will be similar to the cellulose molecule, but with more flexibility due reduce inter-residue hydrogen bond [76].

Additionally, arabinoxylans from cereals have hydroxycinnamic acid substituents, mainly ferulic acid and *p*-coumaric acids, modified on O-5 of some Ara*f* residues [77,78].

The ferulic acid portion can be subject to dimerization by oxidising agents like peroxidase/H_2_O_2_, allowing the formation of a gel-matrix through dimerization of neighbouring arabinoxylan chains [79]. Similar patterns can be adopted by the acetyl ester groups linked to the β-D-xylopyranosyl and α-l-arabinofuranosyl groups [79,80]. In some cereal extract, the arabinoxylan chain was reported with other substituents at the Xyl*p* group, such as glucuronosyl, and 4-*O*-methyl-glucuronosyl residues or short oligomeric side chains (e.g., two or more Ara*f* residues or Ara*f* group with a terminal Xyl*p* group) [81,82].

Barley studies have highlighted the change of substituted to unsubstituted 4-linked xylosyl units in developing coleoptiles from 4:1 to 1:1 [39], indicating that 80% of the *Xylp* residues in the xylan backbone are initially substituted with Ara*f* residues, which are gradually removed during the development [39], by the arabinoxylan arabinofuranohydrolases enzyme [83]. Similar process has been described in maize coleoptiles [84]. Changes in the degree of substitution and in substitution patterns will affect the physicochemical properties of the polysaccharide, such as solubility and its ability to bind to other cell wall components.

Based on other polysaccharides biosynthetic systems, a biological model for the synthesis of the arabinoxylan has been proposed. The mechanism can be divided into four steps: chain or backbone initiation, elongation, side chain addition, and termination and extracellular deposition [85,86]. Type I membrane-bound polysaccharide synthases with multiple transmembrane helices might be expected to mediate the synthesis of the (1,4)-β-xylan backbone, and a type II arabinosyl transferase with a single transmembrane helix might be expected to be involved in the addition of Ara*f* substituents, along with a different type II glucuronyl transferase for the addition of the α-d-glucuronopyranosyl substituents to the (1,4)-β-D-xylan backbone [67].

**Figure 6:**
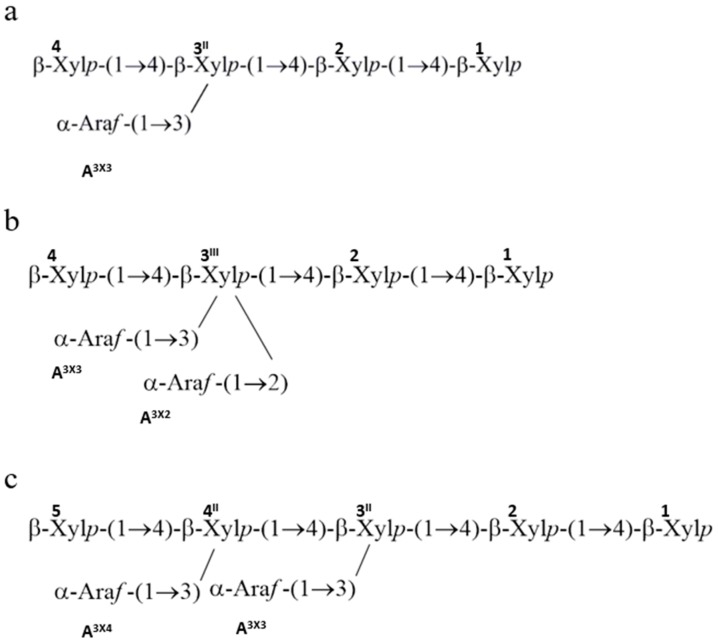
^1^H NMR spectra for oligosaccharides DP5 (**a**), DP6 (**b**) and DP7 (**c**) in durum lines. Figure redrawn from Marcotuli et al., 2016 [95].

Although cellulose synthase-like genes might be predicted as candidate genes for the biosynthesis of (1,4)-β-xylan backbone [26], current evidence has variously implicated GT43, GT47 and GT61 glycosyl transferases in the polymerisation of the xylan backbone chain [87,88,89,90] (Figure 6). Thus, Mitchell et al. [88] identified a group of candidate genes for arabinoxylan biosynthesis in the families GT61, GT47, GT2, CslC, GT43, GT77, GT48 and GT64. Subsequent studies demonstrated that GT61, GT43 and GT47 were associated with the synthesis of arabinoxylan and its side chains [91]. More specifically, GT61 family enzymes are thought to be responsible for the majority of monosubstitution of wheat AX with α-(1,3)-linked Araf [92], while the GT43 and GT47 families located on chromosomes 4 and 3, respectively, are believed to be involved in the synthesis of the AX backbone [93]. Members of the GT47 family, IRX10 and BL29/ESK1 proteins, have shown to be involved in the xylan xylosyltransferase activity, forming β-1,4 xylosidic linkage [94] and in the subsequent addition of O-acetyl groups [95], respectively. Additionally, hydrolytic enzymes (glucosyl hydrolase, GH) could be involved in the biosynthesis of arabinoxylans. In fact, there is good evidence that the fine structure of arabinoxylans changes after the initial deposition of the polysaccharide into walls [25].

## 8. Physio-Chemical and Functional Property of Arabinoxylan

An important indicator of the physio-chemical and functional properties of arabinoxylan is the frequency and allocation pattern of Ara*f* and other groups on the xylan backbone. The Ara*f* and other substituents sterically inhibit aggregation of (1,4)-β-xylan chains, promoting the creation of a wide and asymmetrical polysaccharide in line with its function in the wall of the grasses.

The distribution of the substituent along the arabinoxylan backbone in many cereals seems to be non-random [96,97,98], with some regions characterised by Xyl*p* mono- and di-substitution, others by one to two unsubstituted Xyl*p* residues, and some other with few Ara*f* that are substituents liable to xylanase hydrolysis [99]. These lightly substituted regions would also be amenable to forming intermolecular interactions with other arabinoxylan chains or with other wall polysaccharides, such as cellulose and (1,3;1,4)-β-glucans.

The arabinose-to-xylose (Ara/Xyl) ratio reflects these substitution patterns and therefore is also an indicator of the chain aggregation tendency and consequently solubility. Hence, the high substituted arabinoxylan is more soluble than those with fewer arabinosyl or other substituents. In fact, the water-soluble fraction from wheat arabinoxylan treated with α-l-arabinofuranosidase shows few α-L-arabinofuranosyl units and easily aggregates onto insoluble complexes [100].

The arabinoxylan solubility of cereals is directly connect to health effect in response to many diseases such as colon cancer, glucose tolerance and diverticular diseases [101,102,103,104].

When eaten, this polysaccharide increases the viscosity of the small intestine content and therefore slows down enzyme-mediated starch breakdown. This prolongs food absorption and slows the rate of glucose release after meals, reducing the glycemic index and providing benefit to people with type II diabetes. In the large intestine, this dietary fibre is fermented to various short-chain fatty acids, which suggests it is likely to reduce the risk of colorectal cancer [17]. Dietary fibre also has a significant impact on the health of microbial flora in the human gut and has been recognised as a potential pharmaceutical preventative agent of diet-related chronic diseases, when taken at appropriate doses [105].

Additionally, the exclusive physicochemical properties of arabinoxylan have a significant effect on cereal food industries, as well as bread making [106], fractioning of gluten and starch [107], dough [77,108] and animal feeds [109].

## 9. Cell Walls in Plant–Pathogen Interactions

Cell walls of plants represent one of the first points of contact with invading microorganisms and are often modified by the plant in an attempt to slow or block the penetration of potentially pathogenic bacteria or fungi. Callosic papillae have long been implicated as important deposits that will impede the penetration of invading microorganisms [110]. These papillae contain the (1,3)-β-glucan, callose, as their main polysaccharide component but, in addition, contain arabinoxylans, cellulose and phenolics; most of the latter is probably ferulic acid [111]. Furthermore, genes involved in arabinoxylan biosynthesis influence resistance to fungal penetration in barley [112]. This situation is likely to also occur in durum, where it has been highlighted how xylan arabinosylation explain a protective function against the cell wall from fungal enzymatic degradation [113]. The multiple polysaccharide constituents of callosic papillae provide new targets for the protection of durum against potentially pathogenic microorganisms.

## 10. Arabinoxylan Content and QTL in Durum

Arabinoxylan is a major component of durum endosperm with values ranging from 4% to 9%, determined through reverse phase HPLC as described by Burton et al. [68]. Thanks to the significant variation of arabinoxylan content in different genotypes, QTL analyses have been carried out using a biparental population (both from bread and durum wheat) and tetraploid collections, in order to identify the region controlling the trait.

In Table 3 and Figure 2 all the QTL analyses of durum are reported. In durum, these highlight the differences in number and map position of the marker/trait association identified, due to the different kind of material analysed and the statistical methods applied for the investigation.

Mapping studies for arabinoxylan content identified 31 QTL distributed on all durum chromosomes. Only the QTL on chromosome 1B (at 116.0 cM) has been detected in more than one map/population, indicating the presence of a major QTL associated marker that will be useful for breeding to improve the nutritional value of durum grains. Specific genes associated to the QTL reported in these studies were not identified, with the exception of Marcotuli et al. [27], who were able to identify nine candidate genes associated with regions controlling the trait. These include glycosyl transferase (GT) and glycosyl hydrolase (GH) enzymes, which are coded by genes for the arabinoxylan biosynthesis. In detail, a g*lycosyl hydrolase* and *glycosyltransferase* genes were identified associated to a QTL located on chromosomes 1A, 3A and 5A; on chromosome 2B it was associated with a *Cis-zeatin O-glucosyltransferase-1* gene; a GT1 cluster on chromosome 5A; two members of the GH9 family (1,3-β-D-glucan synthase and β-1,4-endo glucanase) on chromosome 7A.

Although these genes have been identified, no association/mapping studies have detected the main genes involved in the arabinoxylan synthesis.

## 11. Summary and Conclusions

Different advances on non-starch polysaccharides have been reported in the literature on cereal grains.

Understanding the biosynthetic pathway, in fact, is important for the nutritional aspect, food processing activities that include brewing and baking, stock feed manufacture, and protection against pathogenic microorganisms. The knowledge in durum requires many efforts due to the polyploidy and the quantitative nature of these traits.

We report all the significant goals that have been reached in understanding the genetic and molecular mechanisms, and the regulation of (1,3;1,4)-β-glucan and arabinoxylan accumulation in durum kernel. The characterisation of specific plant materials and the release of the durum wheat genome sequences, together with the development of more accurate classes of DNA-based markers and consensus maps, have allowed the identification of important genes involved in the control of (1,3;1,4)-β-glucan and arabinoxylan biosynthesis. Many QTL region have been described to be involved in the control of (1,3;1,4)-β-glucan and arabinoxylan but none of them were associated to one of the *cellulose synthase* (*CslF*, *CslH* and *CslJ*) and *glycosyl transferase* genes (*GT43*, *GT47* and *GT61*), which have been designated as responsible for the regulation and accumulation of (1,3;1,4)-β-glucan and arabinoxylan, respectively, in different tissues types. Nevertheless, the isolation and characterisation of the *CslF6* and *CslH* durum gene sequences have been reported together with the expression pattern in durum endosperm at different developmental stages, increasing the speed of the genetic gains.

The control of these traits by several genes makes it interesting to incorporate beneficial alleles, which can contribute to the rise in non-starch polysaccharides content in durum kernels, into introgressed lines to obtain new durum genotypes with higher (1,3;1,4)-β-glucan and arabinoxylan. The additive effects of some designated genes in the QTL regions reported could be used to generate breeding plants though the marker assisted selection (MAS) approach.

Despite the research in this subject, efforts should be taken to transfer knowledge from barley to durum, in order to get advantages in global breeding programs.

Finally, advanced techniques, such as CRISPR-Cas9-based genome editing method, represent now the new frontiers that should be applied in durum breeding in order to understand how amino acid substitutions or gene silencing can act additively and impose effects on both total gene expression and resulting phenotypes and open a new way for the durum non-starch polysaccharides improvement research.

## Figures and Tables

**Table 1:** Comparison of grain composition of durum and other cereal grains.

Nutrients	*Triticum Turgidum* Durum Wheat	*Triticum Aestivum* Bread Wheat	*Hordeum Vulgare* Barley	*Avena Sativa* Oats	*Oryza Sativa* Rice
Starch (%DM)	71	48	55	44	64
Protein (g)	14	12	13	10	4.4
Fat (g)	2.5	1.7	1.6	1.6	0.4
Dietary fibre (g)	1.6	2.4	10	15	0.6
Thiamin B1 (mg)	0.4	0.1	0.2	0.7	0.1
Riboflavin B2 (mg)	0.1	0.0	0.1	0.1	0.0
Sodium (mg)	3.8	2.0	4.0	2.0	2.0
Potassium (mg)	431	141	309	510	86
Calcium (mg)	34	15	32	24	5.6
Magnesium (mg)	42	13	12	16	34
Iron (mg)	6.8	3.6	2.7	2.0	2.8
Zinc (mg)	4.2	0.7	2	2.1	1.1
Selenium (mg)	70.7	33.9	37.7	-	-

**Table 2:** Summary of QTL for β-glucan reported in wheat from the literature.

Chr	Marker	Map Position	QTL Type	LOD	Encoding Gene	References
1A	IWB42976	10.6	GWAS	3.2	*-*	[47]
1B	Xhbg406	75.0	RIL	3.3	*-*	[56]
2A	IWB66738	11.2	GWAS	3.3	*Starch synthase II*	[47]
2A	IWB1280	47.8	RIL	4.5	*-*	[48]
2A	IWB26593	197.0	GWAS	3.1	*β-amylase*	[47]
2B	IWB30115	2.1	RIL	4.7	*-*	[48]
2B	IWB1898	14.5	GWAS	3.5	*(1,4)-beta-xylanase*	[47]
2B	IWB23783	42.3	RIL	3.8	*β-glucosidase 1a*	[48]
3A	Xbarc45	28.5	RIL	2.8	*-*	[56]
5B	Xgwm540	49.0	RIL	5.3	*-*	[56]
5B	IWB70546	166.0	GWAS	3.2	*-*	[47]
6D	Xcfd80	29.9	RIL	3.1	*-*	[56]
7A	IWB74166	49.7	GWAS	3.4	*Isoamylase*	[47]
7A	IWB68797	90.9	GWAS	3.2	*Fructan 1-exohydrolase*	[47]

**Table 3:** Summary of QTL clusters for arabinoxylan reported in wheat from the literature.

Chrom	Marker	Map Position	QTL Type	LOD	Encoding Gene	References
1A	Y34Ukr-RH13_TOTAX *	-	RIL	3.2	-	[93]
1A	IWA3952	70.1	GWAS	3.1	Glycosyl hydrolase	[27]
1A	IWB64556	113.8	GWAS	3.0	Glycosyltransferase	[27]
1B	Y34Ukr-RH13_TOTAX *	-	RIL	3.2	-	[93]
1B	IWB20653	62.6	GWAS	3.0	-	[27]
1B	IWB8152	110.2	GWAS	3.1	-	[27]
1B	wPt-4129	137.0	RIL	3.9	-	[1]
1B	BA00789946	277.0	RIL	5.1	-	[93]
1B	Xbcd508	116.0	RIL	2.9	-	[2]
1B	bcd508b	116.0	RIL	>2	-	[3]
2A	IWB10662	65.7	GWAS	3.1	-	[27]
2A	wpt-3114	85.7	RIL	22.4	-	[4]
2A	IWB32084	167.9	GWAS	3.0	-	[27]
2B	IWB71648	107.4	GWAS	3.0	Cis-zeatin O-glucosyltransferase 1	[27]
3A	wPt-9422	79.6	RIL	4.1	-	[1]
3A	IWB41859	122.7	GWAS	3.1	Glycosyl hydrolase	[27]
3B	IWB34238	57.2	GWAS	3.0	-	[27]
3D	wpt-0485	0.0	RIL	9.9	-	[4]
4B	IWB71022	55.6	GWAS	3.0	-	[27]
4D	gpw-95001	46.0	RIL	37.7	-	[4]
5A	IWB21456	26.5	GWAS	3.0	Glycosyltransferase	[27]
5A	IWB10271	43.3	GWAS	4.0	Cellulose synthase	[27]
5A	IWB75097	63.7	GWAS	3.0	Glucuronosyltransferase	[27]
6A	IWB2064	126.2	GWAS	3.0	-	[27]
6B	IWB9021	82.6	GWAS	4.0	-	[27]
6B	gwm680	112.4	RIL	9.2	-	[4]
7A	IWB73134	42.1	GWAS	6.1	1,3-β-D-glucan synthase	[27]
7A	IWA2658	130.3	GWAS	3.0	β-1,4-endo glucanase	[27]
7A	IWB34095	179.2	GWAS	3.0	-	[27]
7B	wPt-0963	42.2	RIL	4.0	-	[1]
7B	IWB45276	83.1	GWAS	3.0	-	[27]

* data not published yet.
